# The effects of COVID-19 on self-harm in UK prisons

**DOI:** 10.1192/bjb.2020.83

**Published:** 2020-07-16

**Authors:** Thomas Hewson, Russell Green, Andrew Shepherd, Jake Hard, Jennifer Shaw

**Affiliations:** 1 Manchester University NHS Foundation Trust; 2 CareUK Healthcare; 3 University of Manchester; 4 Royal College of General Practitioners Secure Environments Group

**Keywords:** Coronavirus, COVID-19, self-harm, prison, forensic mental health services

## Abstract

Self-harm is a major international public health concern and is especially prevalent among prisoners. In this editorial, we explore recent trends in prisoner self-harm during the coronavirus lockdown, and consider strategies for improving the prevention and management of self-harm in prisons as we emerge from the pandemic.

The frequency and severity of self-harm have been increasing within UK prisons over recent years, with rates far exceeding those observed in the general population.^1^ We postulated that the coronavirus pandemic could adversely affect the mental health of prisoners and further increase rates of self-harm, given the rapid changes and reductions to prison regimes and the negative psychological effects of quarantine.^2^ However, internal reports from Safer Custody Units in 31 prisons where healthcare is provided by CareUK (R. Green, personal communication, 2020) have revealed fewer implementations of ACCT (assessment, care in custody and teamwork) processes since lockdown; these processes initiate care plans for prisoners at risk of self-harm or suicide. Across the 31 prisons, there were 1079 ACCTs implemented in February 2020, compared with 828 in April 2020, a relative reduction of just under 25%. Furthermore, a closer analysis of eight of these prisons revealed overall reductions in recorded incidents of self-harm, decreasing by a third from 324 in February 2020 to 214 in April 2020. There are many possible reasons for these apparent reductions, with important lessons to be learned.

The accurate recording and maintenance of ACCT processes is a legal requirement stipulated in Prison Service Instructions,^3^ supporting the reliability of the above figures. However, minor acts of self-harm may be more likely to be missed or unrecorded during the pandemic owing to potentially reduced face-to-face contact with prisoners and staffing issues. There have been national reports of fewer Accident & Emergency attendances for various health problems,^4^ raising the possibility that prisoners have similarly been less likely to seek healthcare interventions for self-harm throughout the lockdown. A reduction in ACCTs is also not synonymous with reductions in self-harming behaviour, as multiple factors, including staff discretion, affect ACCT implementation; furthermore, Humber et al found that ACCTs were more likely to be opened following identification of risk factors for self-harm or suicide, rather than following an act of self-harming behaviour.^5^ The data are, however, mirrored by internal reports of recent reductions in prison violence and referrals to mental health teams, as well as fewer calls to see prisoners already known to mental health services (R. Green, personal communication, 2020). These data may not be representative of all prisons, owing to individual differences between establishments, but those included encompassed all security levels and prisoners of varying age, gender and sentence type.

Assuming that self-harm rates have dropped, why might that be and what can we learn for future self-harm risk management? First, although numerous negative psychological consequences are associated with confinement and social isolation,^2^ spending increased time in cells has probably reduced prisoners’ exposure to negative and intimidating behaviours, such as bullying, threats and violence from other inmates. This could increase their overall sense of safety and security. Previously, prisoners may have resorted to self-harm to express their safety concerns, occasionally using this behaviour as a last-resort measure to seek transfer to segregation or another prison.^6^ Furthermore, conflicts with other inmates can cause significant emotional distress, and the most frequently cited reason for self-harm is emotional dysregulation.^7^ Prisoners may be less likely to experience such tensions in the context of reduced peer contact.

Anecdotally, drug use has recently decreased within prisons, probably owing to suspension of prison visits and enhanced difficulties in trafficking illicit substances into custodial settings. There are multiple links between drug use and self-harm, although it is difficult to establish causality.^8^ For example, prisoners may harm themselves while intoxicated because they have less conscious control of their actions, or while withdrawing because of unpleasant physical symptoms. Substance misuse is linked to the aetiology, perpetuation and relapse of various mental disorders, including psychosis, which are associated with increased rates of self-harm and violence. Stress, anxiety and depression may be induced by problems such as accumulation of debt, extortion and violence arising from drug use.^8^ Prisoners may self-harm to communicate distress about these difficulties or to seek escape from them.

The anticipated negative influence of coronavirus on prisoners’ mental health may have encouraged a more pro-active approach among all staff to identify and support prisoners at risk of mental distress. This could have resulted in timely interventions before symptoms escalated to involve self-harm or require high-level input, potentially explaining the apparent decreased number of ACCTs and referrals to mental health teams. Although there have been staffing shortages due to COVID-19, staff who are present may have more time for one-to-one conversation with prisoners, owing to fewer violent incidents and group activities requiring their attention. Staff morale may also be higher owing to greater public recognition of ‘key workers’, which could improve the quality of their interactions with inmates. Furthermore, prisoners may feel more valued, having witnessed various measures being implemented to protect their health. Increased societal cohesion has been observed during prior public health crises, with less differentiation between societal groups and more focus on ‘coming together’ and responding collectively to tackle emergent issues;^9^ anecdotally, this same cohesion has been observed within prisons. Prisoners may also feel better connected to wider society owing to increased communication about external affairs throughout the pandemic. Feeling part of a group is associated with increased self-esteem; particularly among young people, self-harm can be used to achieve a sense of belonging when this is felt to be lacking.^7^

The pandemic has created more of a ‘level playing field’ within prisons, as many of the previously extended rewards for good behaviour are no longer feasible for reasons of infection control, whereas others, such as telephone access, have been made more widely available to increase support for all prisoners during the crisis. This is likely to have reduced feelings of injustice and inequity among prisoners, which can sometimes fuel mental distress and make institutional adjustment more difficult. It could be argued that increasing access to such privileges helps to improve mental well-being; furthermore, it may increase prisoners’ sense of self-worth, an important factor related to self-harm, as those who previously enjoyed fewer rewards may have felt inferior to fellow inmates.

Although prison visits are temporarily suspended, approximately half of English and Welsh prisons have provided secure phone handsets to risk-assessed prisoners,^10^ and some have been trialling virtual family conversations through video platforms; therefore, contact with family and friends may have actually increased for some prisoners, albeit through electronic methods. Such contact can provide much-needed emotional support and act as a protective factor against suicide and self-harm. Increased communication may also alleviate concerns about loved ones and make prisoners feel better connected to outside society. However, the effects of this may vary, and prisoners at the greatest risk of suicide and self-harm, particularly those with serious mental health problems, are more likely to be alienated from support networks; contact with family and friends may be unchanged for these individuals.

Interestingly, suicide rates among the general population have initially dropped during the immediate aftermath of prior national disasters.^11^ This has been attributed to evolving social connectedness and a renewed sense of vigour and purpose, which may shift a person's focus to surviving. Worryingly, this has previously often represented a ‘honeymoon period’, with subsequent increases in suicidality among the general population;^11^ the same may occur with rates of prisoner self-harm. Although there are several possible reasons self-harm may have recently decreased in prisons, there are also multiple mechanisms by which COVID-19 could have profound negative effects on prisoners’ mental health. It is likely that prisons will maintain current reverse cohorting and shielding measures for some time; this is essential to protect against ‘explosive outbreaks’ of the virus, but it further heightens the need for vigilant monitoring of mental well-being, given that prolonged quarantine is associated with poorer psychological outcomes.^2^ Reasons for engaging in self-harm vary widely between prisoners, and changes to prison regimes will likely affect different prisoners differently, depending on individual coping styles, personalities and the presence of pre-existing mental health problems. Prisons must be mindful of these differences and potential future challenges to pre-emptively plan strategies for preventing and treating any future increase in suicidal and self-harming behaviours. Potential difficulties could arise if social distancing measures are eased more quickly in wider society, as this dissonance could reduce prisoners’ sense of ‘social connectedness’ with outside communities and worsen feelings of isolation. Where possible, prisons should coordinate their pandemic responses with external society, ensuring clear communication to prisoners throughout the process.

Multiple measures already exist within prisons to prevent and effectively manage self-harm; examples include ACCT processes, mental health screening and support services, peer support schemes such as ‘Listeners’, and various initiatives for promoting staff understanding of self-harm and positive prisoner–staff relationships.^12,13^ Assuming that the recent reductions in recorded incidents of self-harm and initiations of ACCT processes equate with actual reductions in self-harm in prisons, we must consider what can be learned from the pandemic to improve prisoner safety post COVID-19. The data highlight a need for mental health to be addressed in the prison as a ‘social whole’, with an enhanced focus on preventive social measures to reduce self-harm and creating therapeutic environments; the importance of a whole-prison approach, and of environmental stressors, has been identified previously.^12,13^ A recent rapid evidence assessment identified a lack of research on protective factors for self-harm in prisons;^13^ consequently, researchers, prison staff and inmates need to work together to identify factors helping to reduce self-harm in recent months and how these could be sustained in the future. For example, prisons could consider continuing increased provision of certain ‘privileges’, such as telephone and video communications with external support networks, and must continue implementing strategies to reduce bullying, violence and substance misuse. The increased forms of communication available to prisoners and methods for facilitating in-cell activities should also continue post COVID-19. Importantly, staff must remain alert to any potential future deteriorations in mental health and increases in self-harm throughout the pandemic, ensuring that the negative psychological effects of quarantine are reduced wherever possible, while protection from coronavirus is maintained.

## About the authors

**Thomas Hewson** is an Academic Foundation Doctor at Manchester University NHS Foundation Trust, Manchester, UK. **Russell Green** is National Lead Psychiatrist Health in Justice at CareUK Healthcare (Health in Justice). **Andrew Shepherd** is a Clinical Lecturer in Forensic Psychiatry at the Division of Psychology and Mental Health, University of Manchester, UK. **Jake Hard** is Chair of the Royal College of General Practitioners Secure Environments Group. **Jennifer Shaw** is a Professor of Forensic Psychiatry at the Division of Psychology and Mental Health, University of Manchester, UK, the Greater Manchester Mental Health NHS Foundation Trust, Manchester, UK, and the Independent Advisory Panel on Deaths in Custody, London, UK.
